# Effect of Anaesthesia Depth on Postoperative Delirium and Postoperative Cognitive Dysfunction in High-Risk Patients: A Systematic Review and Meta-Analysis

**DOI:** 10.7759/cureus.30120

**Published:** 2022-10-10

**Authors:** Loni Ling, Timothy Xianyi Yang, Sze Wai Karen Lee

**Affiliations:** 1 Department of Anaesthesiology and Operating Theatre Services, Queen Elizabeth Hospital, Hong Kong, CHN

**Keywords:** elderly population, depth of anesthesia, bispectral index monitoring, postoperative delirium, postoperative cognitive dysfunction

## Abstract

Postoperative delirium (POD) and postoperative cognitive dysfunction (POCD) pose increased morbidity and mortality, especially to elderly patients. The effect of anesthesia is debatable. The databases of PubMed, EMBASE, Google Scholar, Cochrane Library and Web of Science were searched from inception until 24 February 2022 to identify randomized-controlled trials (RCTs) studying the effect of depth of anesthesia on POD and POCD primarily. Data on length of hospital stay and mortality were also extracted. Trial sequential analysis was also performed. Seventeen studies were eligible for systematic review and 15 studies of 5392 patients were eligible for meta-analysis. High bispectral index (BIS) favored a reduction in POD and POCD at three months. We found no significant difference between High BIS and Low BIS for mini-mental state exam (MMSE) score and POCD on day 7. However, this did not translate to a significant difference in length of stay and mortality. The data was also underpowered and heterogeneous. Future RCTs should focus on high-risk patients. A standardized methodology of reporting postoperative delirium and cognitive dysfunction is needed to improve comparisons across trials.

## Introduction and background

With developing countries having an increasing burden of elderly patients, the average age of patients undergoing anesthesia and surgery has increased dramatically. Postoperative cognitive dysfunction (POCD) and postoperative delirium (POD) are more common in the elderly, occurring in up to 65% of older patients [1, 2]. Postoperative cognitive issues in the elderly have become significant issues facing clinicians. POCD and POD are serious postoperative complications in elderly patients, leading to impairments in cognitive function, learning, memory and judgment. These can lead to prolonged hospital stay and increased mortality as well as increased medical costs [1-4]. At least 10% of patients aged over 60 had persistent POCD after three months post-surgery [5].

Multiple risk factors have been identified for POD and POCD. Besides increasing age that we have just mentioned, fewer years of education, depression, and organic brain disease, e.g., lacunar infarct [6-8] are also common risk factors, but these factors are non-modifiable. Anesthetic depth is suspected to be a factor associated with increased POD and POCD. It is a modifiable risk factor that our anesthetic techniques with stringent monitoring can control.

Multiple randomized controlled trials (RCTs) have been performed to assess the effects of anesthesia depth on POCD and POD, but the evidence of the influence of anesthesia technique on POCD and POD is still debatable. Recently, Kunst et al. [9] found bispectral index (BIS) monitoring has significantly reduced postoperative delirium, while no difference in cognitive function at six weeks when compared with the control group without BIS monitoring in elderly patients undergoing coronary artery bypass grafting; Quan et al. [10] showed that deep anesthesia decreased short-term POCD when compared with light anesthesia in patients undergoing abdominal surgery under total intravenous anesthesia; Wildes et al. [11] conducted the ENGAGES trial in elderly patients undergoing major surgery, and comparing EEG-guided group and control group with usual anesthetic care showed that there was no difference in the incidence of POD among the two groups. Multiple meta-analyses were also done, but none have yet included the results of the recent BALANCED study [12] or its sub-study of at-risk individuals [13], which are relatively recent, large RCTs that may add further information. Depth of anesthesia is suspected to be associated with long-term mortality and length of hospital stay as well [14-16].

The present study aimed to assess the relationship between the depth of anesthesia and POD, POCD, mortality, and length of hospital stay. We performed a systematic review of all comparative studies and updated the current literature with a meta-analysis that included the results of the aforementioned study. We evaluated the results with the trial sequential analysis (TSA) method to assess whether the current evidence was conclusive.

## Review

Methods

Search Strategy

The meta-analysis and systematic review followed PRISMA guidelines (Preferred Reporting Items for Systematic Reviews and Meta-Analyses). We searched PubMed, EMBASE, Google Scholar, Cochrane Library and Web of Science from inception until 24 February 2022 for relevant results, using a combination of specific keywords and terms, including “anesthesia depth”, “postoperative delirium” or “POD”, “postoperative cognitive dysfunction” or “POCD”, “BIS” or “bispectral”. We then scanned the reference lists and citations of the studies and any other relevant systematic reviews for further results.

Inclusion and Exclusion Criteria

Strict inclusion and exclusion criteria were applied. For the systematic review, we included all RCTs and well-performed prospective studies on the effects of anesthesia depth on POCD and POD. We only included studies of patients aged over 60. From these studies, we only included the studies where patients were divided into statistically significant High and Low BIS values for the meta-analysis. Only traditionally published journal articles in English were included.

We excluded studies that did not report the primary outcomes, and non-traditional articles including reports, audits, editorials, commentaries, conference reports, and abstracts. Non-English language articles were excluded due to the lack of manpower and budget for professional translation services.

Data Extraction and Quality Assessment

Both authors (Ling and Yang) assessed all the studies independently and evaluated them using the Cochrane Collaboration risk-of-bias tool to assess for selection bias including random sequence generation and allocation concealment, performance bias such as blinding of participants and personnel, detection bias such as blinding of outcome assessment, attrition bias due to incomplete outcome data and reporting bias. Data extraction was also done independently. We categorized the risk of bias as high risk, low risk, or unclear. Disagreements in the process were referred to another investigator (Lee) for review and discussion.

We screened the included studies and extracted the data, and summarized it as a systematic review table. The patient characteristics, study characteristics as well as outcome data were tabulated using standardized forms. The appendices of the studies were checked for any missing data. The authors of the studies were not contacted for missing data due to the risk of introducing bias.

The quality of evidence for each outcome was evaluated by Grading of Recommendations, Assessment, Development, and Evaluations (GRADE) methods and classified as very low, low, moderate, or high [17].

Primary Outcomes

The primary outcomes were the rate of POCD and POD. As both POCD and POD are difficult to define, and different studies used different tests and assessments to define their outcomes, our definitions were based on the individual trials’ definitions.

Secondary Outcomes

The secondary outcomes were mortality and length of hospital stay.

Statistical Analysis

The included studies were screened, and the relevant data were extracted and summarized in a systematic review table. The patient characteristics, study characteristics as well as outcome data were tabulated using standardized forms. The appendices of the studies were checked for any missing data. The authors of the studies were not contacted for missing data due to the potential for introducing bias.

We used the latest version of RevMan (5.4.1) to perform the statistical analysis. As the majority of the randomized controlled trials were small and the degree of heterogeneity was high, a random effects model (DerSimonian and Laird method) was utilized. Pooled estimates for dichotomous outcomes are presented as risk ratios (RRs) with 95% confidence intervals (CIs) and for continuous outcomes, mean differences with standard deviations are used. If the data reports median and interquartile ranges, the data was converted to estimate mean and standard deviation using the method described by Wan et al. [18]. Studies were assessed for heterogeneity using Cochran’s Q test and I^2^ tests. We took I^2^ of 0% to 40% to be insignificant heterogeneity, 41% to 60% to represent moderate heterogeneity, and 61% to 100% to represent substantial heterogeneity. We used funnel plots to assess the risk of publication bias as well as Egger’s regression test. We separated the paper by Valentin et al. [19] into two separate studies as the study authors had four separate arms, High BIS with and without dexamethasone and Low BIS with and without dexamethasone.

We also performed further subgroup analyses to look for confounders, involving subsets of studies, e.g. different surgery types (non-cardiac vs cardiac surgery), different interventions (volatile anesthesia (VA) vs total intravenous anesthesia (TIVA)), as means of investigating heterogeneous results. This acted as an informal test to look for the source of heterogeneity. Note that either the effect or the test for heterogeneity in one subgroup is statistically significant, whilst that in the other subgroup is not statistically significant does not indicate that the subgroup factor explains heterogeneity. Since different subgroups are likely to contain different amounts of information and thus have different abilities to detect effects, it is misleading simply to compare the statistical significance of the results.

We also performed sensitivity analyses to determine any specific sources of heterogeneity. Trial sequential analysis (TSA) was performed to determine if the results of the meta-analysis were reliable, utilizing TSA software version 0.9. Where appropriate, we used a two-sided conventional test boundary assuming a type 1 error of 5% and an alpha-spending O’Brien-Fleming two-sided boundary assuming a type 1 error of 5% and power of 20%, an information axis of sample size, variance-based heterogeneity correction and estimated information size based on empirical risk reduction ratios.

Results

Identification and Characteristics of the Studies

**Figure 1:**
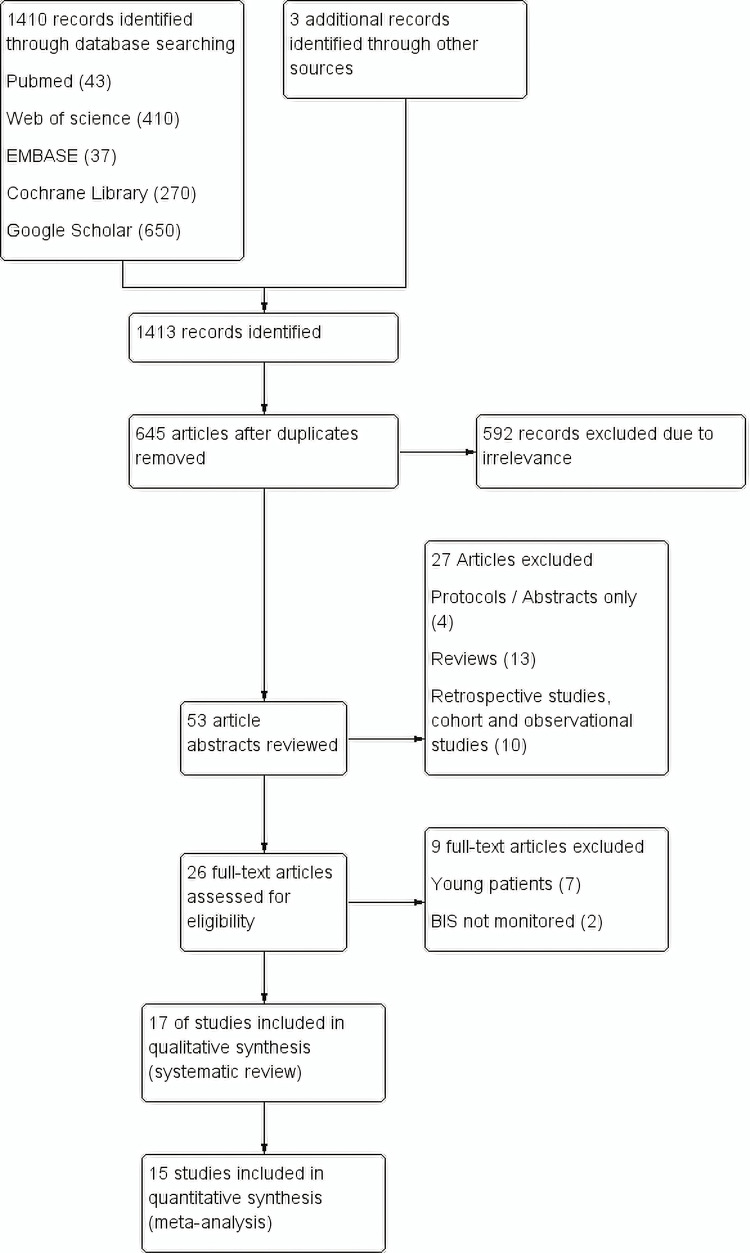
Study flow diagram (PRISMA flow chart)

Figure 1 shows the flow chart of our meta-analysis. A total of 1413 records were identified through database searches and three records through other sources; 645 of which were screened for eligibility after removing duplicates. Fifty-three article abstracts were reviewed after excluding articles of obvious irrelevance. Twenty-six full-text articles were identified after excluding articles of protocols/abstracts, reviews, and retrospective cohorts/observational studies. In the end, 17 studies were included for systematic review, and 15 studies were included in the meta-analysis. Table 1 shows the characteristics of the included studies.

**Table 1:** Characteristics of included studies. BIS: bispectral index; POCD: postoperative cognitive dysfunction; MMSE: Mini-Mental State Exam; SA: spinal anaesthesia; GA: general anesthesia; POD: postoperative delirium; LOS: length of hospital stay; NTI: Narcotrend Index; MAC: minimum alveolar concentration; rScO2: regional cerebral tissue oxygenation; OAA/S: modified observer’s assessment of sedation score; ETAC: End-tidal anesthetic concentration.

Study	Type of surgery	Age (years)	Sample Size	Treatment arms	Outcome
Ballard et al. (2012) [20]	Elective orthopaedic and abdominal surgery	>=60	72	Combined BIS and cerebral oxygenation monitoring vs control	POCD and MMSE at one week, three months and one year
Brown et al. (2021) [21]	Elective spinal surgery	>= 65	217	SA with sedation (targeted BIS) vs GA (masked BIS)	POD, mortality, LOS
Chan et al. (2013) [22]	Elective major surgery	>=60	921	BIS-guided (n=462) vs no BIS-guided (n=459)	POD, POCD at day 7 and three months, mortality, LOS
Chen et al. (2021) [23]	VATS lobectomy	>= 65	73	NTI 50-59 vs NTI 30-39	POD, MMSE
Evered et al. (2021) [13]	Major surgery	>=60	515	High BIS 50 (n=253) vs Low BIS 35 (n=262)	POD, MMSE
Hou et al. (2018) [24]	Elective total knee replacement	>=60	60	High BIS 55-65 (n=30) vs Low BIS 40-50 (n=30)	POCD at day 7 and three months
Kunst et al. (2019) [9]	Elective coronary artery bypass graft surgery on cardiopulmonary bypass	>=65	82	BIS and rScO2-guided (n=42) vs BIS and rScO2-blinded (n=40)	POD, MMSE, LOS
Quan et al. (2019) [10]	Abdominal surgery	>=65	120	Low BIS 30-45 (n=60) vs High BIS 45-60 (n=60)	POCD at day 7 and three months, mortality, LOS
Radtke et al. (2013) [25]	Abdominal, thoracic, vascular, orthopaedic, otorhinolaryngological, oral and maxillofacial, gynaecological, and urologic surgery	>=60	1155	BIS-guided (n=575) vs BIS-blinded (n=580)	POD, POCD at day 7 and three months, morality, LOS
Sadek et al. (2010) [26]	Elective spine surgery	>=60	40	BIS-targeted (50-55) (n=20) and MAC-targeted (n=20)	MMSE
Sieber et al. (2010) [27]	Hip fracture repair	>=65	114	BIS 50 (n=57) vs BIS>=80 (n=57)	POD, MMSE, mortality, LOS
Sieber et al. (2018) [28]	Non-elective hip fracture repair	>=65	200	OAA/S Heavier (modified observer’s assessment of sedation score of 0-2) (n=100) or lighter (observer’s assessment of sedation score of 3-5) (n=100)	POD, MMSE, LOS
Valentin et al. (2016) [19]	Elective non-cardiac and non-neurologic surgery	>=60	140	control + low BIS 35-45 (n=40) vs control + high BIS 46-55 (n=32) vs dexamethasone + low BIS (n=36) vs dexamethasone + high BIS (n=32)	POCD at day 7 and three months
Whitlock et al. (2014) [29]	Cardiothoracic patients	>60	310	BIS vs ETAC	POD
Wildes et al. (2019) [11]	Major surgeries (e.g., cardiac, gastrointestinal, thoracic, gynecologic, hepatobiliary-pancreatic, urologic, vascular)	>=60	1232	BIS-guided (n=614) vs usual care (n=618)	POD, mortality, LOS
Wong et al. (2002) [30]	Elective knee or hip replacement surgery	>=60	60	BIS group (n=29) vs standard practice group (n=31)	MMSE
Zhou et al. (2018) [31]	Resection of colon carcinoma	65-75	81	BIS group (n=41) vs non-BIS group (n=40)	POD

Risk of Bias

**Figure 2:**
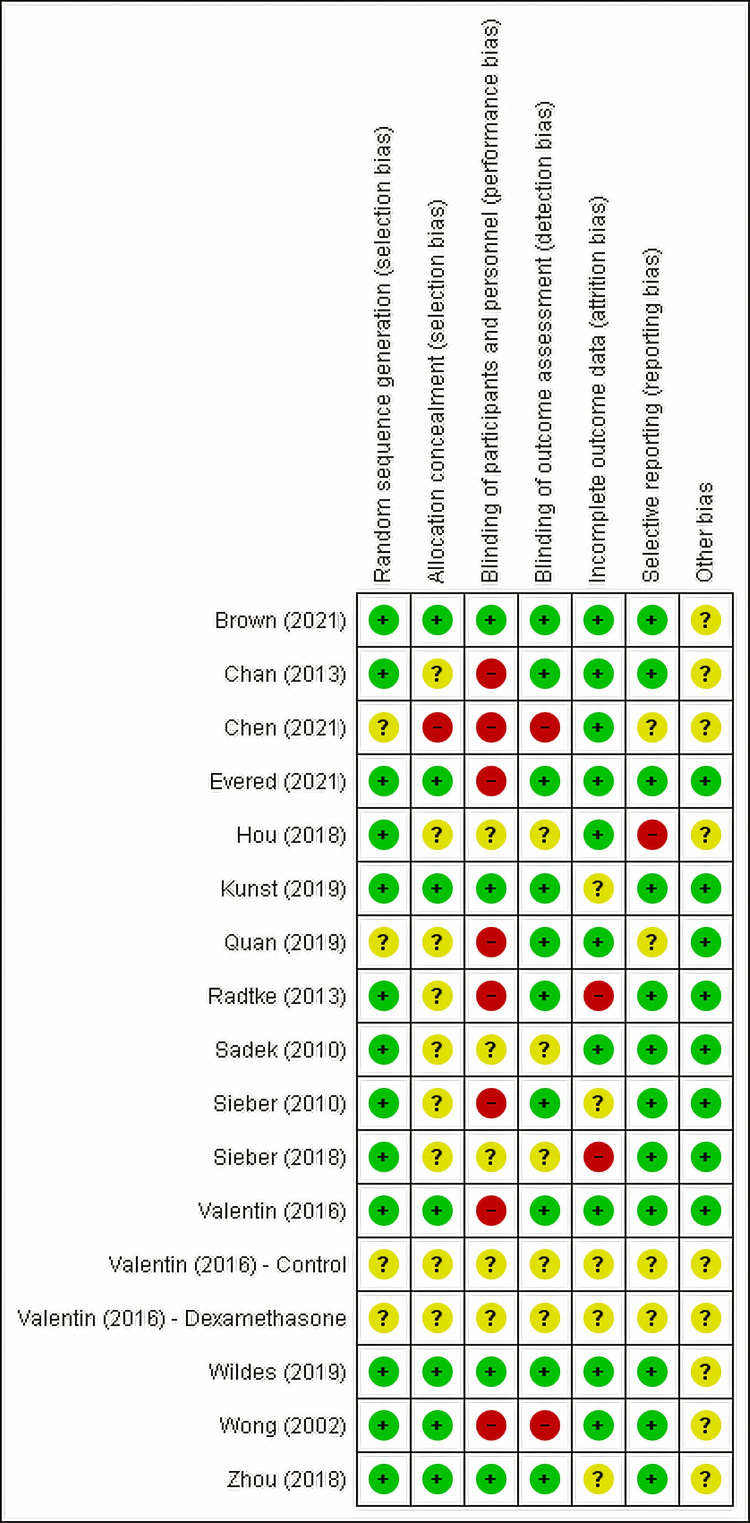
Risk of Bias Summary “+”: low risk of bias; “?”: unclear risk of bias; “-”: high risk of bias.

**Figure 3:**
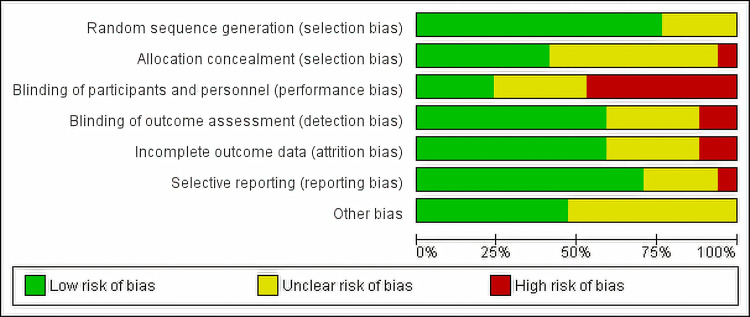
Risk of Bias Graph

Most studies were judged to be low to unclear risk of bias. The risk of bias of the included RCTs was summarized in Figure 2 and Figure 3.

Postoperative Delirium

**Figure 4:**
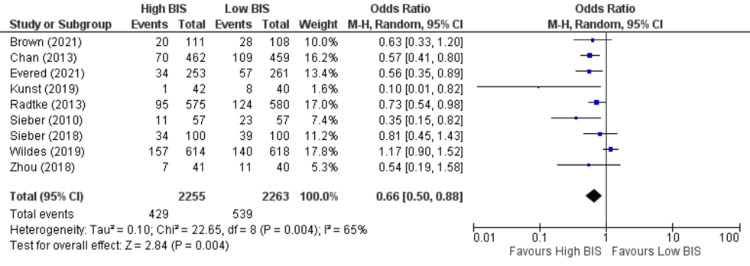
Forest Plot of Postoperative Delirium (POD). BIS: bispectral index; CI: confidence interval. Brown [21], Chan [22], Evered [13], Kunst [9], Radtke [25], Sieber [27], Sieber [28], Wildes [11], Zhou [31].

**Figure 5:**
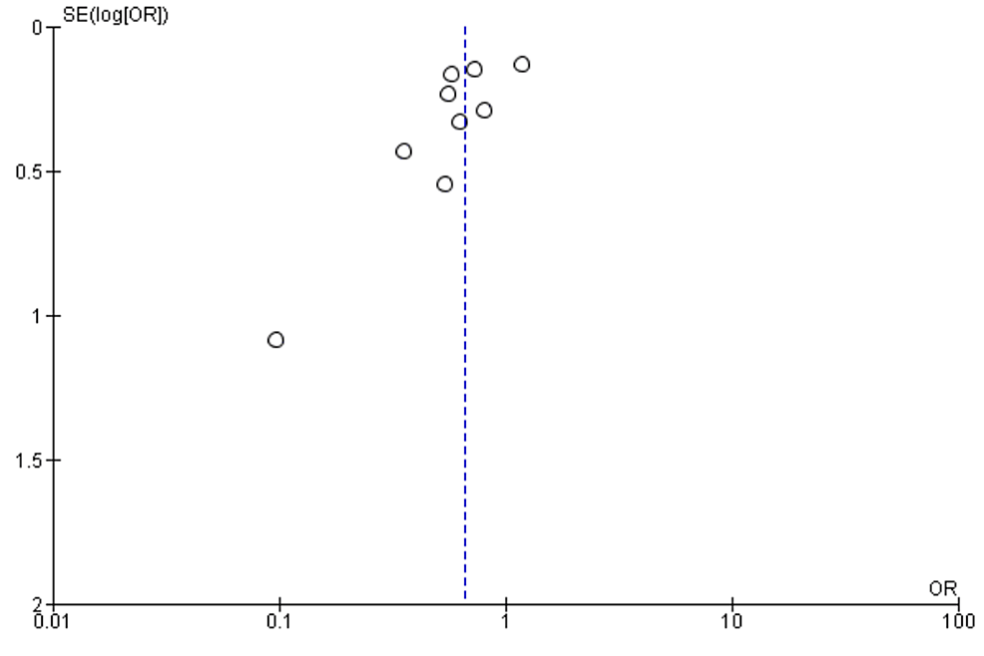
Funnel Plot of Postoperative Delirium (POD)

Nine studies [9,11,13,21,22,25,27,28,31] reported POD as an outcome, including 4518 patients. We found High BIS favored a reduction in POD as reported in Figure 4, [OR 0.66, 95% CI (0.50 to 0.88)]. However, there was substantial heterogeneity with an I^2^ of 65% and a significant Z-value (p = 0.004). Visual examination of the funnel plot (Figure 5) found no evidence of publication bias. Sensitivity analysis was performed, and no individual study was found to be the source of the heterogeneity. Subgroup analysis of only non-cardiac surgeries was performed with a similar outcome [OR 0.69, 95% CI (0.53 to 0.90), I^2^ = 63%, Z value (P = 0.007)]. Subgroup analysis of TIVA vs VA studies found similar outcomes [OR 0.64, 95% CI (0.50 to 0.82), I^2^ = 4%, Z value (P = 0.0003) for VA vs OR 0.70, 95% CI (0.51 to 0.96), I^2^ = 73%, Z value (P = 0.0.03) for TIVA].

Mini-Mental State Examination Score on Postoperative Day Three

**Figure 6:**
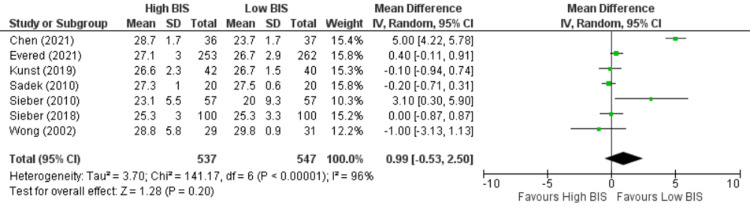
Forest Plot of MMSE on postoperative day three. MMSE: mini-mental state examination; BIS: bispectral index; CI: confidence interval. Chen [23], Evered [13], Kunst [9], Sadek [26], Sieber [27], Sieber [28], Wong [30].

**Figure 7:**
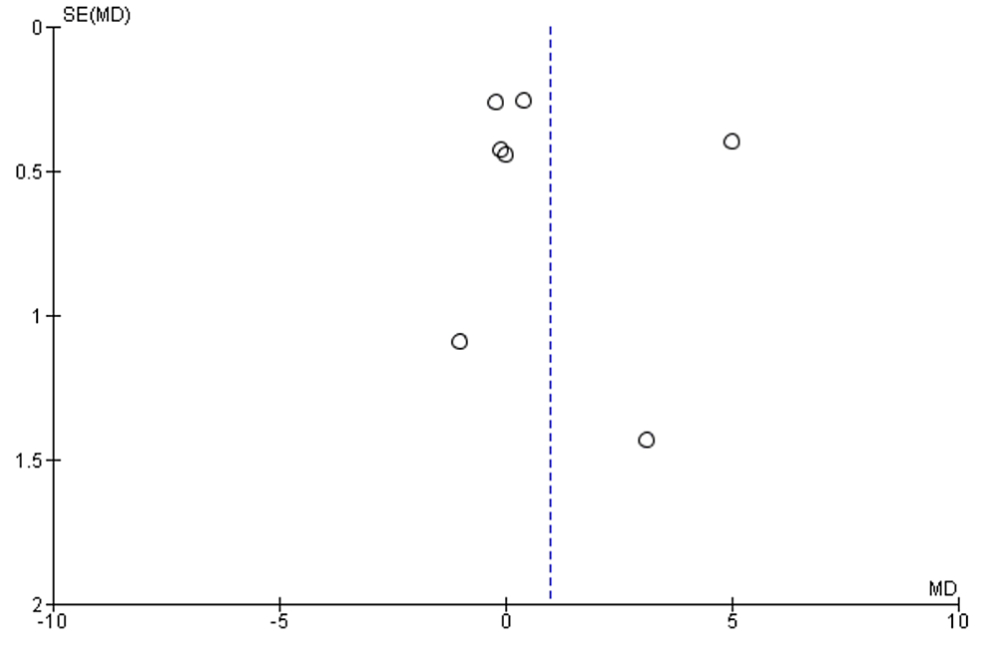
Funnel Plot of MMSE on postoperative day three. MMSE: mini-mental state examination

The included studies used different psychological tests to assess POCD, and MMSE was one of the most commonly used tests in all the studies. Seven studies [9,13,23,26-28,30] reported MMSE on postoperative day 3, including 1084 patients. We found no significant difference between High BIS and Low BIS as reported in Figure 6 [OR 0.99, 95% CI (-0.53 to 2.50)]. There was significant heterogeneity with an I^2^ of 96%. The Z-value was insignificant (p = 0.20). Visual examination of the funnel plot (Figure 7) found no evidence of publication bias. Sensitivity analysis was performed, and Chen (2021) [23] was found to be the source of significant heterogeneity. However, the results remained unchanged, and there was no significant difference between High and Low BIS without Chen (2021) [OR 0.08, 95% CI (-0.37, 0.53), I^2^ = 40%, Z value (p = 0.74)]. Subgroup analysis of only non-cardiac surgeries was performed with a similar outcome [OR = 1.18, 95% CI (-0.58 to 2.95), I^2^ = 96%, Z value (P = 0.19)].

Postoperative Cognitive Dysfunction on Postoperative Day Seven

**Figure 8:**
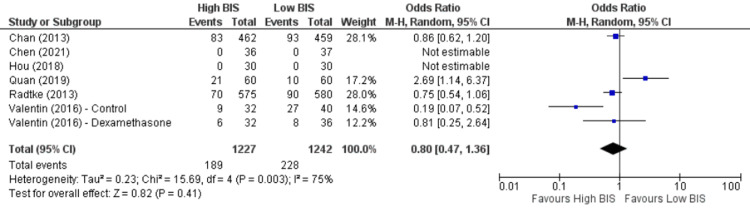
Forest Plot of POCD on postoperative day seven. BIS: bispectral index; CI: confidence interval; POCD: postoperative cognitive dysfunction. Chan [22], Chen [23], Hou [24], Quan [10], Radtke [25], Valentin [19].

**Figure 9:**
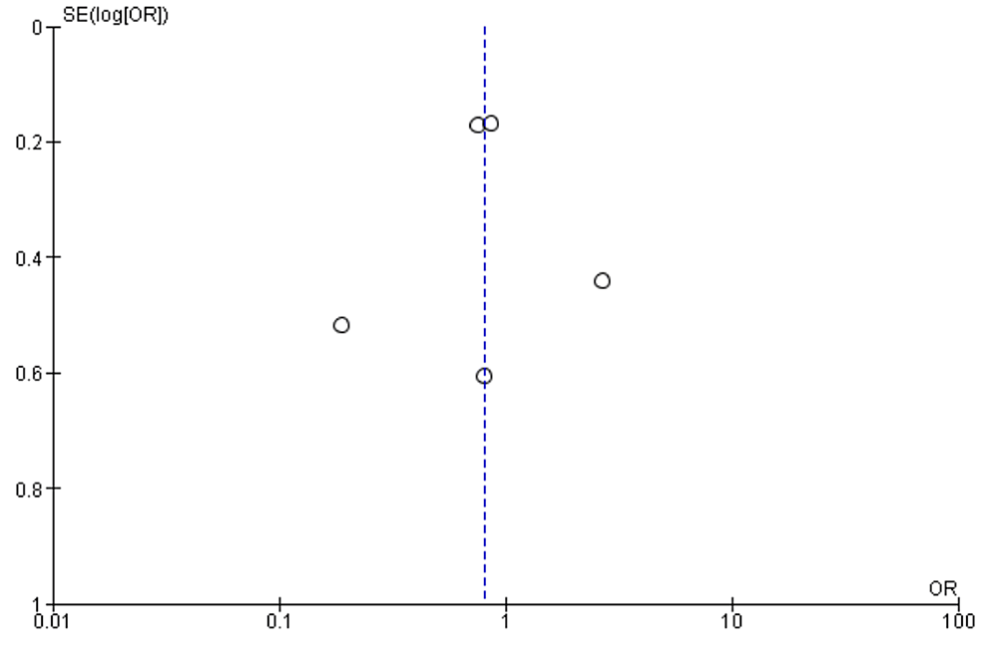
Funnel Plot of POCD on postoperative day seven. POCD: postoperative cognitive dysfunction.

Seven studies [10,19,22-25] reported POCD on postoperative day seven, including 2469 patients. We found no significant difference between High BIS and Low BIS as reported in Figure 8, [OR 0.80, 95% CI (0.47 to 1.36)]. There was substantial heterogeneity with an I^2^ of 75%. The Z-value was insignificant (p = 0.41). Visual examination of the funnel plot (Figure 9) found no evidence of publication bias. Sensitivity analysis was performed, and Quan (2019) [10] was found to be the source of heterogeneity. On removal of Quan (2019), we found a reduction of heterogeneity (I^2^ = 62%, Z value (P = 0.07)], and a substantial change in the outcome [OR = 0.66, 95% CI (0.42 to 1.03)]. Subgroup analysis was not performed as all the included studies were non-cardiac surgery.

Postoperative Cognitive Dysfunction at Three Months

**Figure 10:**
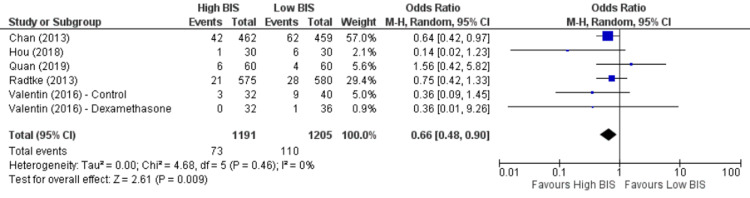
Forest plot of POCD at three months. BIS: bispectral index; CI: confidence interval; POCD: postoperative cognitive dysfunction. Chan [22], Hou [24], Quan [10], Radtke [25], Valentin [19].

**Figure 11:**
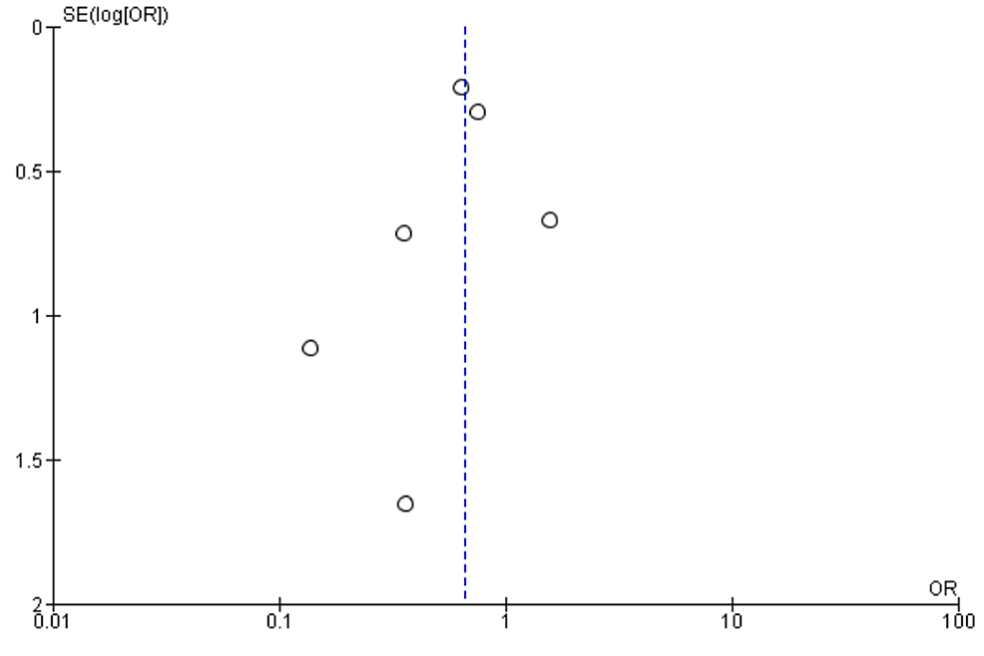
Funnel Plot of POCD at three months. POCD: postoperative cognitive dysfunction.

Five studies [10,19,22,24,25] reported POCD at three months, including 2396 patients. We found High BIS favored a reduction in POCD at three months as reported in Figure 10 [OR 0.66, 95% CI (0.48 to 0.90)]. There was insignificant heterogeneity with an I^2^ of 0%. The Z-value was significant (p = 0.009). Visual examination of the funnel plot (Figure 11) found no evidence of publication bias. Sensitivity analysis was performed and no individual study was found to be the source of the heterogeneity. Subgroup analysis was not performed as all the included studies were non-cardiac surgery.

Mortality

**Figure 12:**
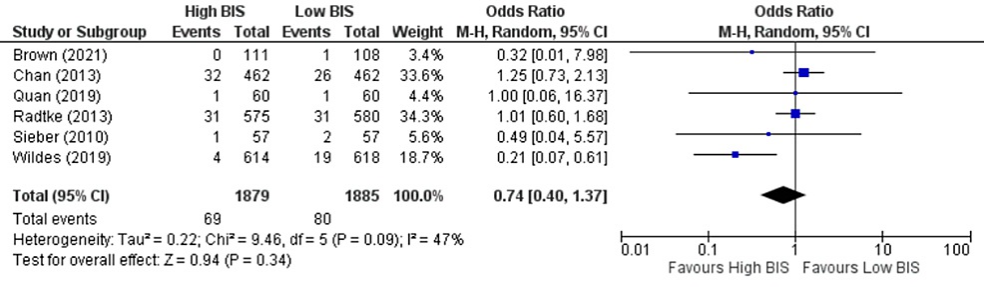
Forest Plot of Mortality. BIS: bispectral index; CI: confidence interval. Brown [21], Chan [22], Quan [10], Radtke [25], Sieber [27], Wildes [11].

**Figure 13:**
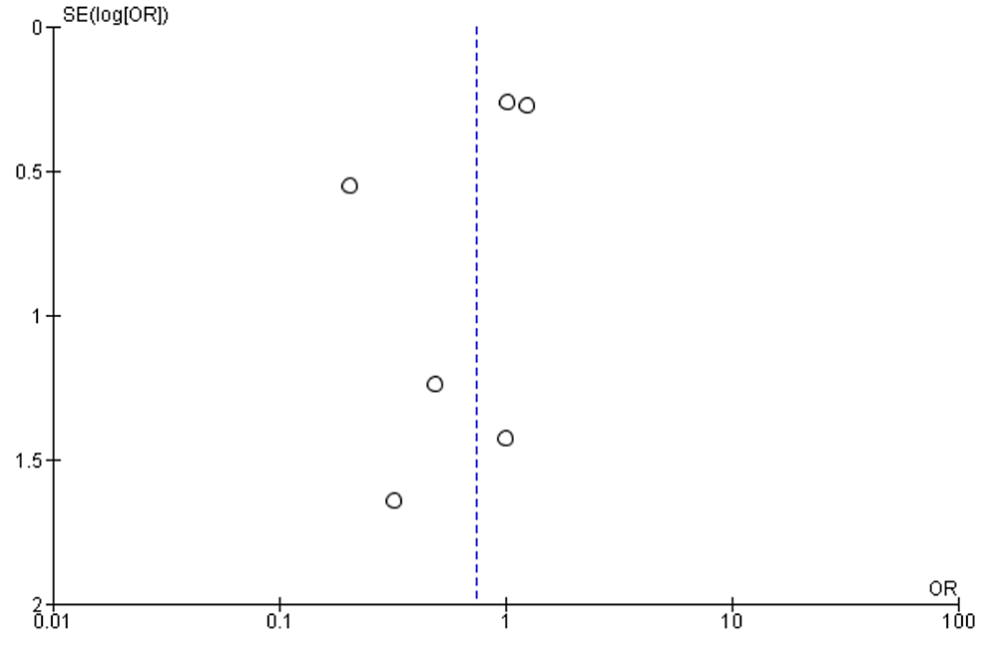
Funnel Plot of Mortality.

Six studies [10,11,21,22,25,27] reported mortality, including 3764 patients. We found no significant difference between High BIS and Low BIS as reported in Figure 12, [OR 0.74, 95% CI (0.40 to 1.37)]. There was moderate heterogeneity with an I^2^ of 47%. The Z-value was insignificant (p = 0.34). Visual examination of the funnel plot (Figure 13) found no evidence of publication bias. Sensitivity analysis was performed, and Wildes (2019) [11] was found to be the source of the heterogeneity. On removal of Wildes (2019), heterogeneity was reduced to I^2^ = 0%, with insignificant Z-value (p = 0.68), but the outcomes remained unchanged [OR 1.08, 95% CI (0.75 to 1.55)]. Subgroup analysis was not performed as all the included studies were non-cardiac surgery.

Length of Stay

**Figure 14:**
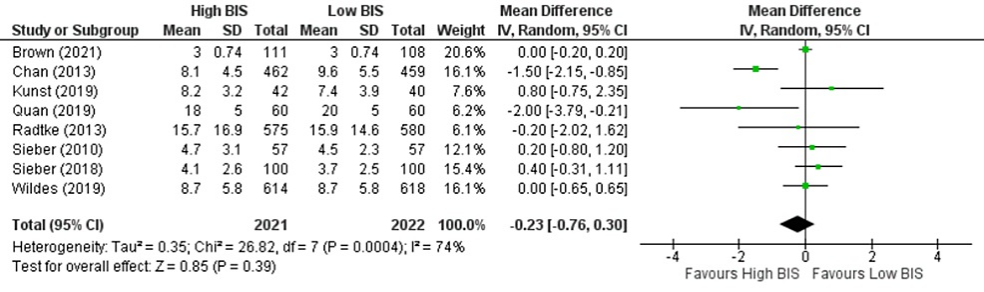
Forest plot of Length of Stay. BIS: bispectral index; CI: confidence interval. Brown [21], Chan [22], Kunst [9], Quan [10], Radtke [25], Sieber [27], Sieber [28], Wildes [11].

**Figure 15:**
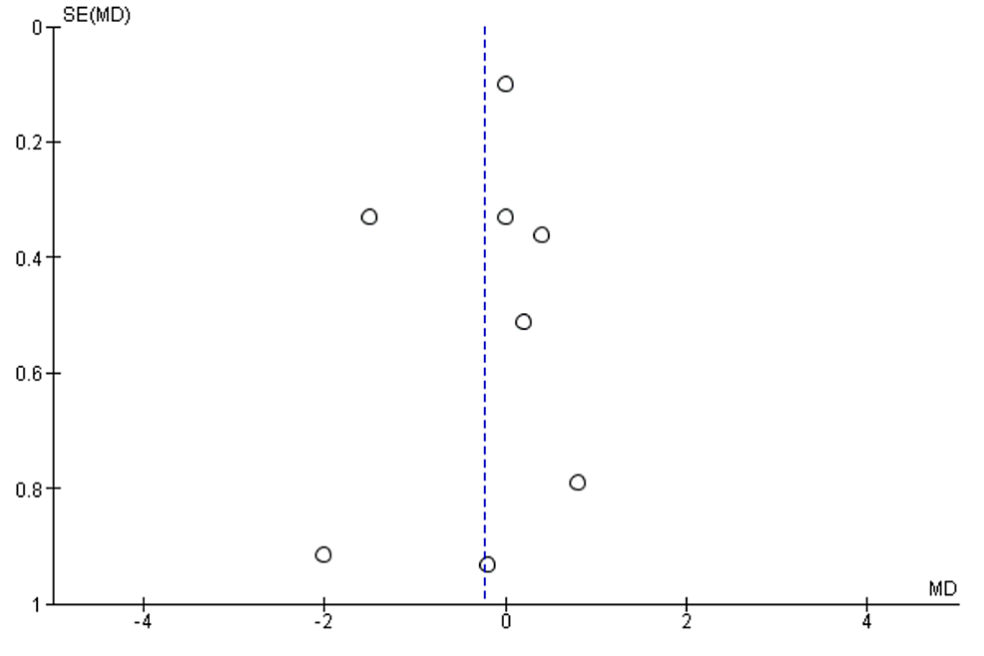
Funnel Plot of Length of Stay.

Eight studies [9-11,21,22,25,27,28] reported length of stay including 4043 patients. We found no significant difference between High BIS and Low BIS as reported in Figure 14 [OR -0.23, 95% CI (-0.76 to 0.30)]. There was substantial heterogeneity with an I^2^ of 74%. The Z-value was insignificant (p = 0.39). Visual examination of the funnel plot (Figure 15) found no evidence of publication bias. Sensitivity analysis was performed, and Chan (2013) [22] was found to be the source of the heterogeneity. On removal of Chan (2013), heterogeneity was reduced to I^2^ = 17%, with insignificant Z-value (p = 0.78), but outcomes remained the same [OR 0.04, 95% CI (-0.24, 0.33)]. Subgroup analysis was not performed as all the included studies were non-cardiac surgery.

Trial Sequential Analysis

**Figure 16:**
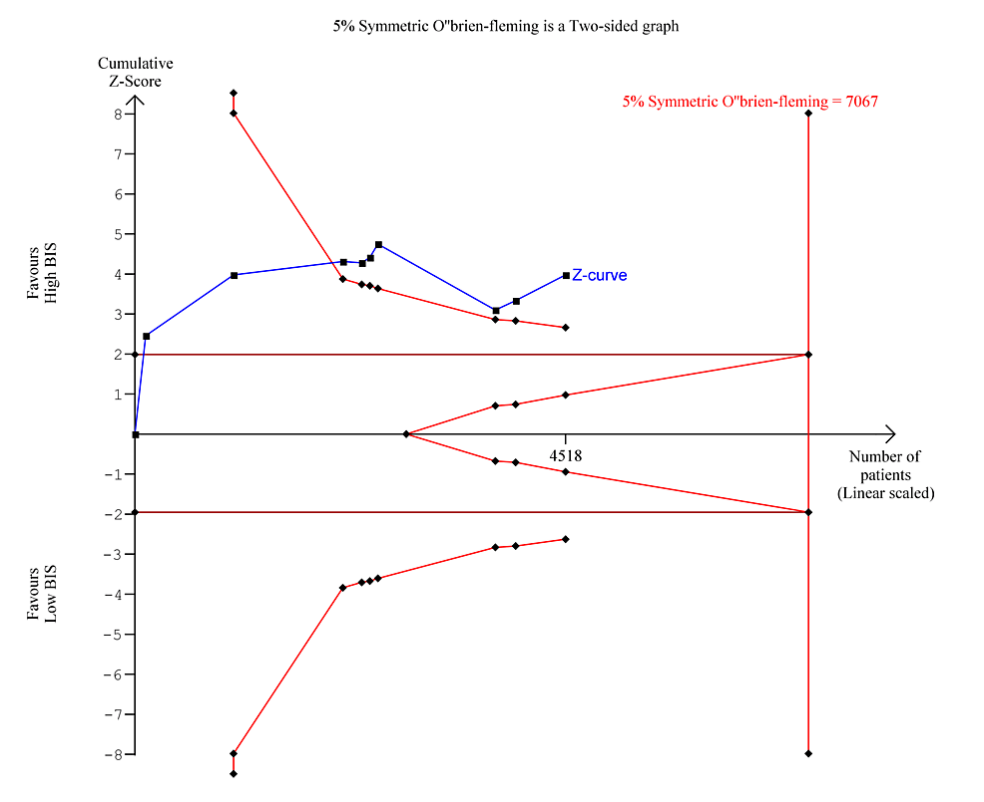
Trial Sequential Analysis for Post Operative Delirium. Y-axis: the cumulative Z-Score; Horizontal green dotted lines: conventional boundaries (upper for benefit, Z-score = 1.96, lower for harm, Z-score = −1.96, two-sided P = 0.05); Sloping red full lines with black square fill icons: trial sequential monitoring boundaries calculated accordingly; Blue full line with black square fill icons: Z-curve; Vertical red full line: required information size calculated accordingly.

**Figure 17:**
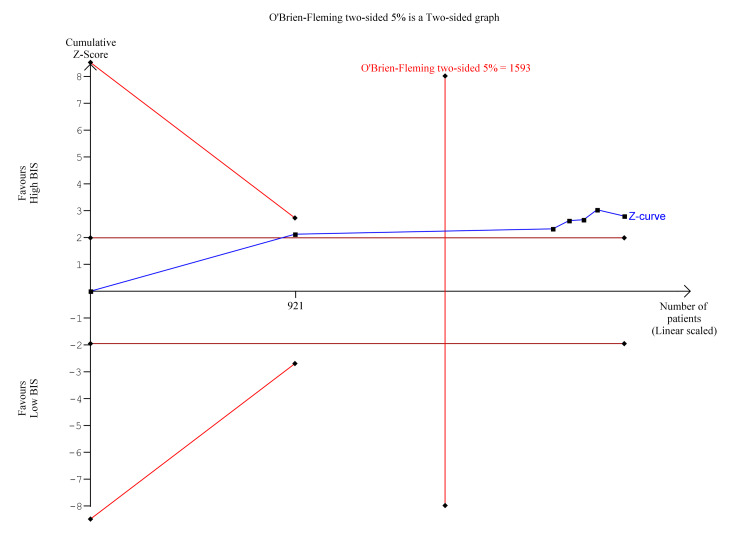
Trial Sequential Analysis for Postoperative Cognitive Dysfunction at three months. Y-axis: the cumulative Z-Score; Horizontal green dotted lines: conventional boundaries (upper for benefit, Z-score = 1.96, lower for harm, Z-score = −1.96, two-sided P = 0.05); Sloping red full lines with black square fill icons: trial sequential monitoring boundaries calculated accordingly; Blue full line with black square fill icons: Z-curve; Vertical red full line: required information size calculated accordingly.

Trial sequential analysis is a methodology used in meta-analyses to control random errors and assess the need for further trials, similar to interim analyses performed in single trials used to decide whether a trial should be terminated early because of a sufficiently small P-value. Trial sequential analysis was performed for the significant findings of postoperative delirium and POCD at three months. As seen in Figure 16, for postoperative delirium, the Z-curve (blue line) crossed the boundary for conventional benefit as well as the monitoring boundary which indicates that High BIS was likely beneficial for reducing postoperative delirium. However, the estimated information size was not reached due to insufficient statistical power. In Figure 17, for POCD at three months, the z-curve crosses the boundary for conventional benefit and monitoring boundary, and exceeds the information size, indicating that High BIS was beneficial for reducing POCD at three months.

Discussion

In this meta-analysis, we showed that there were protective effects of High BIS value on postoperative delirium and postoperative cognitive dysfunction at three months; however, TSA showed that the result for POD was underpowered, while POCD at three months was statistically significant.

Nine meta-analyses [32-40] were found studying the effect of BIS monitoring on postoperative delirium and postoperative cognitive dysfunction, but our study has included more randomized controlled trials than most of them and included the most recent RCT of Evered et al. [13] while being more stringent on patient population and intervention of study. Bocskai et al. [32] also showed protective effects of BIS monitoring on POD on day 1 and POCD at 12 weeks, but relatively fewer studies were included, and younger patients with lower risk for POD and POCD were also included. Oliveira et al. [33] showed significantly reduced incidence of POD and POCD at 12 weeks, but only included studies of Chan (2013) [22] and Radtke (2013) [25]. MacKenzie et al. [34] and Shan et al. [35] only did meta-analyses on POD and BIS monitoring was found to be protective. The meta-analysis of Li and Zhang [36] was done in 2020 with similar papers included in our study, and also showed significantly reduced incidence of POD, POCD at day 1 and day 90 by BIS monitoring; however, they did not include Evered (2021) [13] and study which used auditory evoked potential (AEP) as the depth of anesthesia monitoring was also included (Jildenstål 2011 [41]). We excluded Jildenstål (2011) because AEP values could not be directly converted to BIS values. Two other meta-analyses (Lu (2018) [39] and Miao (2019) [40]) showed no correlation between BIS monitoring and postoperative neurocognitive dysfunction (NCD); however, Lu et al. included only very few studies and study sizes were small, and although Miao et al. also included only high-risk elderly patients, the study included heterogeneous neurocognitive evaluation which would have caused the biased incidence of postoperative NCD.

Meta-analyses on observational studies [14,16] showed that deep anesthesia was associated with long-term mortality. RCTs included in our study failed to show any significance; also, variable time points for reporting mortality were noted. Some studies [42,43] found an association between postoperative delirium and longer length of hospital stay, but in our study, we were unable to show a correlation between depth of anesthesia and length of hospital stay.

Our study included randomized controlled trials exclusively and also included the subset study of the recent large RCT (Evered (2021) [13]), which had not yet been included in any published meta-analyses. POCD can occur in any age group [1], but the elderly patients have lower cognitive reserve and POCD would lead to a significant change in the quality of life and pose a greater burden on the medical system. Our study was stringent on the patient population and only included studies with patients older than 60 years of age. The quality of the evidence was assessed using trial sequential analysis and the GRADE framework.

There are several limitations of our study. Our data showed high heterogeneity. For most RCTs, postoperative delirium was assessed by CAM or CAM-ICU score or psychological tests according to DSM-IV criteria; data could be easily analyzed. However, postoperative cognitive dysfunction had various tests for assessment. Some RCTs only reported preoperative and postoperative MMSE scores, while some used a battery of neuropsychological tests and reported absolute change in results without defining POCD through the results. Time intervals for follow-up were also variable. Therefore fewer data could be extracted from the RCTs when assessing POCD. Also, more RCTs are required to perform subgroup studies on different types of surgeries; more stringent protocols are required to minimize confounding factors, e.g., hypotension, intraoperatively.

In 2017, the European Society of Anaesthesiology published a guideline on postoperative delirium [44]. It recommended monitoring the depth of anesthesia intraoperatively for the prevention of postoperative delirium. The grade of recommendation was “strong” and it was based on papers that were included in our study. Also, the most recent guideline from the Association of Anaesthetists “Recommendations for standards of monitoring during anaesthesia and recovery 2021 [45]” also recommended the use of processed electroencephalogram (pEEG) for monitoring the depth of anesthesia. It stated that pEEG would not only avoid inadequate doses causing accidental awareness but also would avoid excessive doses, which may cause POD and POCD. The results of our study further support the routine use of pEEG, especially on advanced-age patients.

Our study only reviewed the effect of the depth of anesthesia on the incidence of POD and POCD. Other modifiable anesthetic factors, including the type of anesthesia, and different anesthetic drugs, may also pose effects on POD and POCD. Some studies were done to compare the incidence of POD and POCD among the use of propofol, benzodiazepines, or dexmedetomidine, but the results were conflicting [46-48]. A lot of studies reviewed the incidence of POD and POCD between general anesthesia and regional anesthesia, but the results were still debatable [49-51]. More studies are required to study the effect of anesthesia on POD and POCD, in order to minimize the impact of anesthesia on patients.

## Conclusions

There is more evidence showing that postoperative delirium and cognitive dysfunction are significant in the elderly after anesthesia, and they pose a detrimental effect on the quality of life of these patients. Depth of anesthesia is one of the potentially modifiable risk factors to minimize the effect of anesthesia on patients’ cognitive function. Our meta-analysis showed that a high BIS value favored less postoperative delirium and postoperative cognitive dysfunction at three months in elderly patients, but this did not translate to a significant difference in length of stay and mortality. Future RCTs should focus on high-risk patients, and a standardized methodology of reporting POD and POCD is needed to improve comparisons across trials.
